# Oral health-related quality of life and patient-reported outcome measures after 10 years of supportive periodontal care

**DOI:** 10.1007/s00784-023-04876-9

**Published:** 2023-02-01

**Authors:** Luca Vogt, Bernadette Pretzl, Peter Eickholz, Tatjana Ramich, Katrin Nickles, Hari Petsos

**Affiliations:** 1 Private practice, Hundemstraße 4, 57368 Lennestadt, Germany; 2 Dental Academy, Lorenzstraße 7, 76135 Karlsruhe, Germany; 3 Ruprecht-Karls-University Heidelberg, 69117 Heidelberg, Germany; 4 Department of Periodontology, Center of Dentistry and Oral Medicine (Carolinum), Goethe-University Frankfurt, Theodor-Stern-Kai 7, 60596 Frankfurt/Main, Germany; 5 Private practice, An der Stuferklinik 2, 73557 Mutlangen, Germany; 6 Private practice, Talstraße 1a, 68259 Mannheim, Germany; 7 Private practice, Schloßstraße 25, 35510 Butzbach, Germany

**Keywords:** Periodontitis, Supportive periodontal care, Patient-reported outcome measures, Oral Health Impact Profile (OHIP), Oral health-related quality of life

## Abstract

**Objective:**

The aim of this retrospective study was to evaluate the oral health-related quality of life (oHRQoL) and patient-reported outcome measures (PROMs) after 10 years of supportive periodontal care (SPC).

**Material and methods:**

Patients were re-examined 120±12 months after active periodontal therapy. Dental and periodontal status and oHRQoL by completing Oral Health Impact Profile-G49 (OHIP-G49) and PROMs by marking a visual analogue scale (VAS) for self-perceived esthetics (VASe), chewing function (VASc), and hygiene ability (VASh) were assessed. Patient- and tooth-related factors (age, insurance status, number of SPC, compliance, change of therapist, smoking, tooth loss, need for surgery or antibiotic intake, bleeding on probing (BOP), periodontal inflamed surface area) influencing oHRQoL and PROMs were evaluated.

**Results:**

One hundred eight periodontally compromised patients (59 female, mean age 65.4±10.7 years) lost 135 teeth during 10 years of SPC. At re-examination, 1.8% of all sites showed PPD ≥6mm. The mean OHIP-G49 sum score was 17.6±18.5, and VAS resulted in 76.0±22.5 (VASe), 86.3±16.3 (VASc), and 79.8±15.8 (VASh). Linear regression analyses identified a positive correlation with oHRQoL and/or PROMs for private insurance status (OHIP-G49, *p*=0.015, *R*^2^=0.204; VASc, *p*=0.005, *R*^2^=0.084; VASh, *p*=0.012, *R*^2^=0.222) and compliance to SPC (VASe, *p*=0.032; *R*^2^=0.204), as well as a negative correlation for active smoking (VASc, *p*=0.012, *R*^2^=0.084), increased BOP (VASh, *p*=0.029, *R*^2^=0.222) at the start of SPC, and number of lost molars (VASh, *p*=0.008, *R*^2^=0.222).

**Conclusion:**

It is realistic to obtain satisfactory oHRQoL and PROM values in most of the patients after 10 years of SPC. The identified factors may help to predict patient satisfaction in the long-term course of therapy.

**Clinical relevance:**

Systematic therapy of periodontally compromised patients provides values for oHRQoL and PROMs in a favorable range 10 years after therapy. This should encourage dentists to implement SPC in their daily routine.

**Clinical trial number:**

NCT03048045

## Introduction

Periodontitis is a chronic inflammatory multifactorial disease associated with dysbiotic biofilm and characterized by progressive destruction of the periodontium [1]. It is one of the most common chronic diseases affecting more than 65% of the population worldwide [2]. If left untreated, periodontitis can influence not only clinical parameters such as bleeding on probing (BOP), periodontal probing depth (PPD), and clinical attachment level (CAL), but may also lead to a significant reduction of oral health-related quality of life (oHRQoL) [3]. Moreover, specific patient-reported outcome measures (PROMs) like patient’s satisfaction, function, and esthetics are key features of the perceived state of periodontal disease [4, 5]. Therefore, a systematic evaluation of the patients’ level of satisfaction may help to improve dental care and to promote a joint decision-making process [6–8].

However, in general dental practice, poor confidence in and low perceived utility of periodontal therapy for severely compromised teeth are a common finding [9]. A documentation of a high long-term patient satisfaction that may be achieved after systematic periodontal therapy in a consistently periodontally treated cohort could confirm existing studies and contribute to turning the previously mentioned low level of trust of dental professionals in periodontal therapy into a motivation to carry it out. In addition to the tooth-preserving effect, this would also have a cost-reducing effect, as has been shown in numerous studies [10–12]. Moreover, recently published systematic review with meta-analysis on the recurrence and progression of periodontitis as part of the EFP S3 level clinical practice guideline for treatment of stage IV periodontitis recommends reporting PROMs [13, 14]. Despite increasing call for patient-centered treatment, there is often a lack of standardized assessment of patient satisfaction in associated studies [15, 16]. This results in little evidence on the long-term development of oHRQol and PROMs after periodontal treatment, which is why the present study aims to expand evidence [17–20].

The standardized Oral Health Impact Profile (OHIP) questionnaire is a widely used tool to assess and quantify oHRQoL. In this context, the OHIP-49 questionnaire aims to evaluate the social implications of oral diseases [21]. To be able to objectively record the subjective feeling of patients with regard to specific questions (PROMs), the visual analogue scale (VAS) is a regularly used tool [22].

The aim of this retrospective cohort study was to document the long-term impact of periodontitis treatment and adherence to SPC on oHRQoL and patient satisfaction and to compare them with the existing scientific evidence.

## Material and methods

Most patients’ patient- and tooth-related data used in this study have already been published elsewhere [23, 24].

### Patients and systematic periodontal treatment

The present study was approved by the Institutional Review Board for Human Studies of the Medical Faculty of Johann Wolfgang Goethe-University Frankfurt am Main (approval number: 61/15), conducted in accordance with the 1975 Declaration of Helsinki as revised in 2013, and registered in the clinical trials database of the US National Library of Medicine (ID: NCT03048045). All patients gave written informed consent to participate in this study.

Patients have been identified by electronic and manual database searches based on dental billing items, using the following inclusion criteria:

- Systematic periodontal therapy after April 2004 (introduction of a new therapeutic concept by the newly appointed Head of the Department, PE) at the Department of Periodontology of the Johann Wolfgang Goethe-University Frankfurt am Main.
- Complete periodontal status (PPD and CAL at 6 sites per tooth and furcation involvement (FI) [25, 36], tooth mobility) at:

1. T0: prior to the start of therapy
2. T1: after the end of active periodontal therapy (APT) (non-surgical/step 1 and 2 as well as, if necessary, surgical treatment/step 3 [26]) and at the start of SPC (T1) after re-evaluation
3. T2: 120±12 months after T1

- All participants had to be at least 18 years old (T2).

After initial oral hygiene instruction and supragingival dental debridement, the therapeutic concept included subgingival instrumentation as a modification of the full-mouth disinfection (FMD) concept [27, 28]. Microbiological testing was conducted in aggressive and generalized severe chronic periodontitis prior to treatment [29]. In case of detection of *Aggregatibacter actinomycetemcomitan*s, FMD was combined with adjunctive systemic antibiotic regimen (amoxicillin 500mg and metronidazole 400mg 3× daily, in case of intolerance to amoxicillin, ciprofloxacin 250mg and metronidazole 500mg 2× daily for 1 week). Periodontal surgery was performed for sites exhibiting PPD ≥ 6mm after FMD [23, 24]. 120±12 months after completion of APT (T1), patients were consecutively recruited until ≥ 100 patients could be followed-up.

### Clinical examination and patient records

The number of teeth was documented at the different examination time points (T0, T1, T2). Based on periodonal records at T0, all patients were divided into different stages according to the 2018 classification of periodontal diseases based on interdental CAL, periodontitis-related tooth loss and complexity [30]. The indices collected during SPC (gingival bleeding index (GBI) [31], plaque control record (PCR) [32]) were extracted from patient records. The individual periodontitis risk was determined using the periodontal risk assessment (PRA) model [33] to calculate the respective SPC interval prospectively in each individual session [34, 35]. To determine compliance, the recommended SPC intervals were compared to the actual intervals documented in the patient records. If a patient exceeded the interval once by more than 100%, she/he was classified as non-compliant [34]. In addition, the insurance status and the number of changes of therapist (at least 1× in 10 years) were documented.

#### Supportive periodontal care

Patients were moved to SPC if at most of isolated PPD>6mm were present, which, according to the decision of the respective dentist and patient, did not require any further surgical treatment and could have been kept stable within the framework of regular SPC. All SPCs were performed in a university setting by dentists together with dental assistants/dental hygienists or students under supervision of dentists. A standardized diagnosis and treatment regimen was followed throughout the process [23, 24, 34]:

1.GBI [31] and PCR [32]

2.Establishment of effective individual plaque control through re-instruction and re-motivation

3.Professional mechanical plaque removal (PMPR)

4.Fluoridation (Elmex Gelée; GABA Switzerland AG, Therwil, Switzerland)

5.Dental status and complete periodontal status (PPD, CAL, BOP, FI, and tooth mobility). If PPD = 4mm + BOP or PPD ≥ 5mm, re-instrumentation was performed, and 1% chlorhexidine digluconate gel (Chlorhexamed, GlaxoSmithKline GmbH) was instilled.

Patient files were checked, if teeth were removed in the center or in the authors department, to verify reasons such as periodontal (combination of progressive CAL-V loss, furcation involvement II/III [25], and/or tooth mobility II/III [36]), caries/endodontic (carious lesions that could not be restored, endodontic complications that could not be managed by revision), orthodontic (lack of space, balancing extractions), prosthodontic reasons (unusable as an abutment tooth), or trauma (longitudinal untreatable fractures). Patients who lost teeth outside of the center were asked about the respective reason.

If a patient had >5 teeth with PPD ≥ 5mm 2 years after completion of APT, an additional systematic periodontal therapy (step I/II, if necessary, step III [26]) was recommended.

#### Ten-year follow-up

Ten years after completion of APT, patients were re-examined by four different therapists (KN, TR, PE, HP) (T2) [23, 24]:

1.Self-reported smoking status (non-smoker (never smoked), former smoker (quit smoking ≥5 years ago), and active smoker (quit smoking <5 years ago or current smoker) [33])

2.Medical history

3.Dental status

4.GBI [31] and PCR [32]

5.PPD and CAL with 1.0 mm accuracy using a manual, millimeter-scale rigid periodontal probe (PCPUNC 15, Hu-Friedy, Chicago, USA) at 6 sites per tooth and BOP 30 s after obtaining probing parameters

6.FI on all multi-rooted teeth with Nabers probe (PQ2N, Hu-Friedy) [25]

9.Self-reported marital status (with or without partner)

10. Self-reported educational status (low, primary; middle, secondary, apprenticeship; high, upper secondary)

All therapists involved were experienced periodontists having completed their postgraduate training for at least 3 years and had been calibrated for PPD and CAL among themselves as part of various projects [23, 24, 37, 38].

### Oral Health Impact Profile (G49)

The OHIP-G49 questionnaire includes 49 questions addressing seven subscales: functional limitations, physical pain, psychological discomfort, physical disability, psychological disability, social disability, and handicap [39, 40]. The German version of the OHIP-G49 questionnaire comprises another subscale with 4 additional questions; however, for reasons of international comparability, this subscale was not included in the calculation of the sum score. The frequency of the experienced impairments is categorized by means of a Likert scale ranging from 0 to 4 (0 = never; 1 = almost never; 2 = occasionally; 3 = quite often; 4 = very often) and recorded in the OHIP questionnaire [39]. Each patient completed the questionnaire independently during the 10-year follow-up examination prior to the clinical examination [41].

If more than five questions were not answered, or if more than two answers were missing within one group, the questionnaire was considered invalid [39]. However, a statistical evaluation was carried out also in cases in which 1–4 questions of different subscales remained unanswered [42, 43]. The questionnaire was given to the patients at the beginning of the follow-up examination. The clinical parameters were collected and discussed afterwards with the patients.

### Visual analogue scale

Using a VAS, patients were asked to express their subjective perception of their own perceived esthetics (VAS esthetic, VASe), chewing function (VAS chewing, VASc), and hygiene ability (VAS hygiene, VASh) by drawing a vertical line through the 100-mm-long horizontal VAS scale. The marked value was then measured using a ruler and transferred to a data matrix [44]. Similar to the OHIP-G49 values, the VAS values were also collected prior to the clinical examination.

### Statistical analysis

All data on oHRQoL and PROMs were entered into an Excel-based data matrix (Excel version 16.23; Microsoft, Redmond, USA) by an examiner who was blinded with regard to the clinical results (LV). The primary target parameters were the OHIP-G49 sum score and the VAS values (VASe, VASc, VASh). Third molars were not included in the tooth-related analysis.

Descriptive data were calculated for categorical variables using absolute and relative frequencies. Metric variables were described using mean, standard deviation, median, interquartile range, and minimum/maximum. Tooth-related data were described separately at T0, T1, and T2.

For OHIP-G49, VASe, VASc, and VASh, a bivariate Spearman rank correlation with the corresponding patient- and tooth-related parameters (gender, age, smoking status, insurance status, initial diagnosis, number of SPCs, compliance, change of therapist, BOP, PCR, number of teeth, loss of anteriors/premolars/molars, total tooth loss, need of surgery/antibiotic intake/recurrence therapy, PPD/CAL frequency, PISA) was calculated, and only those showing a significant (*p*<0.05) correlation were included in the respective linear regression analysis. For OHIP-G49, VASe, VASc, and VASh as dependent variables, a separate linear regression analysis was performed. The adjusted *R*^2^ was calculated to describe the quality of the model.

A significance level of 0.05 was assumed. All statistical analyses were performed with appropriate software (SPSS Statistics 24 software package; IBM, Chicago, USA).

## Results

### Patient characteristics

**Fig. 1:**
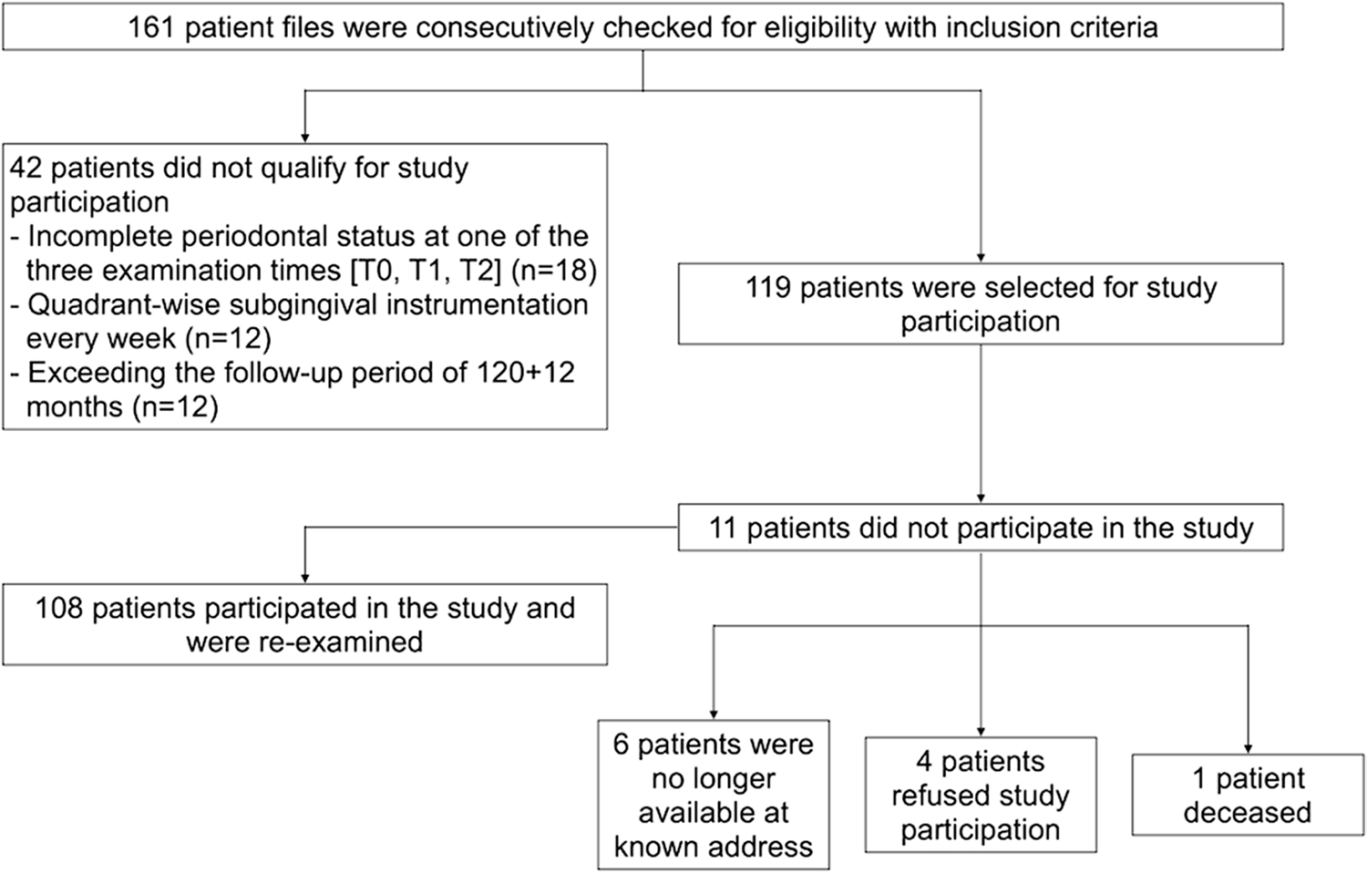
Patient flowchart

Of the original 161 patients who underwent screening, 108 patients were included in our study (Fig. 1). Approximately half of these patients (54.6%) were female. The Patients’ mean age at the start of SPC (T1) was 55.2±10.8 years. Eighty-six patients (79.6%) were non-smokers, 7 (6.5%) former smokers, and 15 (13.9%) active smokers. At start of treatment, 23 study participants suffered from localized stage III, 54 from generalized stage III, and 25 from stage IV, while 6 patients exhibited a molar incisor pattern. Nearly half of the study participants (47.2%) were privately insured. During APT, 15 patients were treated with systemic antibiotics, and 51 underwent surgery after SI. Twelve patients (11.1%) required recurrence therapy during SPC. More than half of the patients (56.4%) were compliant. During the 10-year period, 68 patients changed their therapist at least once. More detailed patient-related data are depicted in Table 1.

**Table 1:** Patient characteristics

	*n*	%	Significant correlation with oHRQoL and/or PROMs
Gender (female/male)	59/49	54.6/45.4	–
Age at start of SPC [years]			D
Mean ± SD	55.2 ± 10.8		
Median (IQR)	55.5 (47.0/64.0)		
Smoking status at start of SPC			C, D
Non-smoker	86	79.6	
Former smoker	7	6.5	
Active smoker	15	13.9	
Antibiotics during APT	15	13.9	D
Surgery during APT	51	47.2	D
SPC			
Number/patient			A, B, D
Mean ± SD (range)	22.6 ± 9.0 (2–41)		
Median (IQR)	21.5/17.0/30.0)		
Compliance			A, B, D
Compliant	61	56.4	
Non-compliant	47	43.4	
Change of therapist (yes/no)	68/40	63.0/37.0	A, B
Initial diagnosis			D
Localized stage III	23	21.3	
Generalized stage III	54	50.0	
Stage IV	25	23.1	
MIP	6	5.6	
Insurance status			A, C, D
Statutory	57	52.8	
Private	51	47.2	
Marital status*			–
Single	17	16.0	
With partner	67	63.2	
Widowed/divorced/separated	22	20.8	
Educational status**			–
Low	0	0.0	
Moderate	36	33.6	
High	71	66.4	

*SD*, standard deviation; *IQR*, interquartile range; *SPC*, supportive periodontal care; *APT*, active periodontal therapy; *MIP*, molar–incisor–pattern; PROMs, patient-reported outcome measures

*Two patients declined to supply the information

*One patient declined to supply the information

Significant bivariate correlation (Spearman rank correlation, *p* < 0.05) with OHIP-G49 sum score (A), VAS for self-perceived esthetic (B), VAS for chewing function (C), and/or VAS for hygiene ability (D)

### Therapy-related results and tooth loss

Of a total of 2586 teeth present at T1, 135 teeth (0.12±0.17 per patient/year) were lost during SPC, and approximately half of them (47.4%) were molars. Most teeth were lost due to periodontal (34%), conservative (32%), or prosthodontic (25%) reasons (Table 2). Compared to baseline (T0), a decrease in the percentage of PPD ≥ 4mm in favor of shallow PPD < 4mm was observed at both T1 and T2. The same applied to the CAL values. Most of the patients exhibited tooth- or implant-supported fixed partial dentures at the time of follow-up examination. For more detailed treatment-related results, see Table 3.

**Table 2:** Tooth loss during SPC

Total tooth loss (*n*)	135
Per patient (mean ± SD/median/IQR)	1.25 ± 1.74 (0.0/0.0; 2.0)
Per patient per year (mean ± SD/median/IQR)	0.12 ± 0.17 (0.0/0.0; 0.2)
Anteriors (*n*)	30
Premolars (*n*)	41
Molars (*n*)	64^D^
Reasons for tooth loss during SPC (n)	
Periodontal	46
Caries/endodontic	43
Orthodontic	5^C,D^
Prosthodontic	34
Vertical fractures	7

*SD*, standard deviation; *IQR*, interquartile range; *SPC*, supportive periodontal care

Significant bivariate correlation (Spearman rank correlation, *p* < 0.05) with OHIP-G49 sum score (A), VAS for self-perceived esthetic (B), VAS for chewing function (C), and/or VAS for hygiene ability (D) marked with superscript letter

**Table 3:** Therapy-related outcomes

	T0 mean ± SD (median/ IQR)	T1 mean ± SD (median/ IQR)	T2 mean ± SD (median/ IQR)
Teeth			
Total (*n*)	2626	2586	2451
Per patient	24.3 ± 3.9 (25.0/ 23.0; 27.0)	23.9 ± 4.1 (25.0/ 23.0; 27.0)	22.7 ± 5.9 (23.5/ 20.3; 26.0)
Restorations (patients, *n*)			
Fixed tooth-supported restorations	72	72	53
Fixed implant-supported restorations	15	18	40
Removable restorations	2	3	5
PPD (sites, %)			
< 4 mm	69.9 ± 15.4 (73.3/ 61.2; 81.5)	88.0 ± 8.5 (89.5/ 83.5; 93.9)	88.8 ± 10.4 (90.4/ 84.6; 96.6)
4–5 mm	20.1 ± 9.6 (18.7/ 13.1; 26.0)	10.5 ± 7.1 (9.2/ 5.0; 14.1)	9.4 ± 8.2 (8.1/ 3.0/ 13.7)
> 5 mm	10.0 ± 9.6 (7.7/ 2.5; 14.0)	1.5 ± 2.1 (0.7/ 0.0; 2.3)	1.8 ± 3.1 (0.6/ 0.0; 2.1)
Mean PPD (mm)	3.25 ± 0.62 (3.19/ 2.77; 3.54)	2.50 ± 0.34 (2.50/ 2.25; 2.71)	2.45 ± 0.41 (2.40/ 2.20; 2.67)
CAL (sites, %)			
< 4 mm	56.5 ± 22.0 (59.8/ 41.5; 72.6)	66.9 ± 22.3 (71.9/ 54.7; 84.5)	66.9 ± 22.5 (71.5/ 55.1; 83.8)
4–5 mm	27.5 ± 13.2 (26.1/ 16.8; 37.0)	24.0 ± 14.5 (21.8/ 11.3; 33.2)	23.5 ± 13.6 (23.0/ 13.2; 33.2)
> 5 mm	16.0 ± 14.5 (12.7/ 5.5; 20.1)	9.1 ± 10.9 (4.9/ 1.4; 11.5)	9.6 ± 12.4 (5.2/ 1.3; 13.6)
Mean CAL (mm)	3.76 ± 0.92 (3.66/ 3.16; 4.15)	3.23 ± 0.81 (3.07/ 2.74; 3.58)	3.25 ± 0.87^D^ (3.10/ 2.69; 3.63)
Furcation involved teeth (n)			
FI I	311^A^	316^C,D^	337
FI II	174	107	74
FI III	80	52	53
Mobile teeth (n)			
Degree I	475^A^	310	137
Degree II	167	68	33^B^
Degree III	50	20	13^B^
PISA	397.0 ± 377.1 (313.8/ 83.0; 650.3)	184.3 ± 148.8 (145.1/ 72.2; 255.4)^C, D^	247.9 ± 192.7 (201.5/ 96.3; 321.7)
BOP (%)	26.4 ± 20.9 (21.5/ 12.9; 33.5)	13.2 ± 8.9 (11.5/ 7.0; 18.8)^C, D^	18.2 ± 11.8 (16.0/ 9.0; 24.8)
PCR (%)	- (*)	30.2 ± 17.6 (27.5/ 16.3; 39.8)^A^	33.2 ± 18.4 (31.0/ 19.3; 46.0)

*SD*, standard deviation; *IQR*, interquartile range; *SPC*, supportive periodontal care; *PPD*, periodontal probing depths; *CAL*, clinical attachment level; *FI*, furcation involvement; *PISA*, periodontally inflamed surface area; *BOP*, bleeding on probing; *PCR*, plaque control record

*PCR is not documented before start of active periodontal treatment

Significant bivariate correlation (Spearman rank correlation, *p* < 0.05) with OHIP-G49 sum score (A), VAS for self-perceived esthetic (B), VAS for chewing function (C), and/or VAS for hygiene ability (D) marked with superscript letter

### oHRQoL and PROMs

Due to a transcription error made at the start of the study, three questions from three different subscales were missing in all OHIP-G49 questionnaires:

1. “In the past months, did you suffer from indigestion that might have been caused by problems with your teeth, oral area or dental prosthesis?”
2. “In the past months, did you suffer from pain in the gums?”
3. “In the past months, have you been completely uncapable of doing anything at all because of problems with your teeth, oral area, or dental prosthesis?”

As a consequence, the arithmetic mean of the respective OHIP sheet was used to compensate for the missing data [43].

**Fig. 2:**
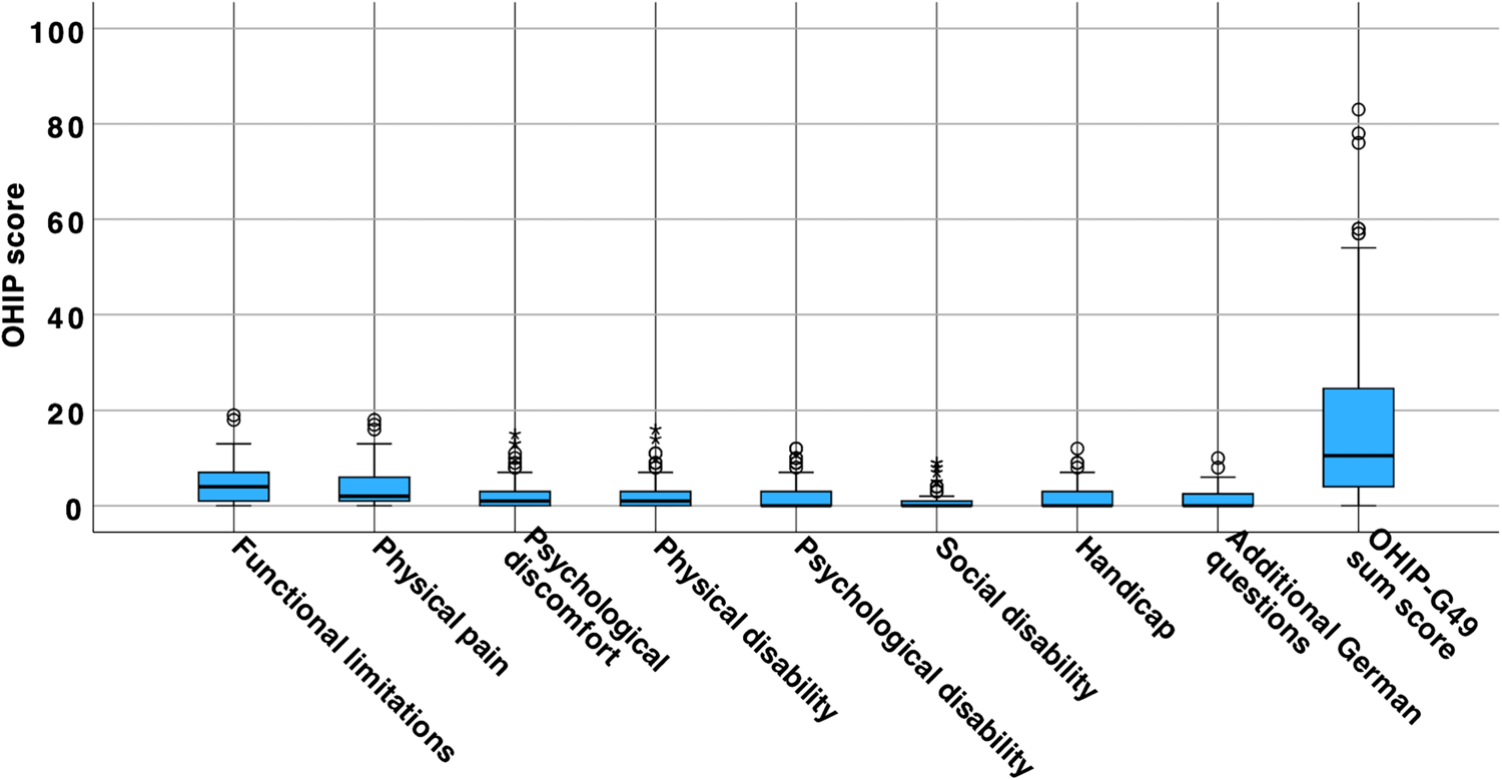
Distribution of OHIP-G49 sum score and subscale ratings (wide black line, median; box, 25–75% range of all values; whiskers, range of all values without outliers; circle, outliers; asterisk, extreme)

PROMs are depicted in Table 4. The mean OHIP sum score was 17.6±18.5. The mean score was the highest in the subscale “functional limitations” (4.4±3.8) and the lowest in the subscale “social disability” (1.2±2.1). Overall, all subscales as well as the sum score displayed a high frequency of low OHIP values (Fig. 2).

**Table 4:** Oral health-related quality of life and patient-reported outcome measures

	Median (IQR)	Mean ± SD (range)
OHIP-G49		
Functional limitations	4.0 (1.0/7.0)	4.4 ± 3.8 (0–9)
Physical pain	2.0 (1.0/6.0)	3.9 ± 4.1 (0–18)
Psychological discomfort	1.0 (0.0/3.0)	2.2 ± 3.2 (0–15)
Physical disability	1.0 (0.0/3.0)	2.4 ± 3.3 (0–16)
Psychological disability	0.0 (0.0/3.0)	2.0 ± 3.1 (0–12)
Social disability	0.0 (0.0/1.0)	1.2 ± 2.1 (0–9)
Handicap	0.0 (0.0/3.0)	1.6 ± 2.5 (0–12)
Additional German questions	0.0 (0.0/2.75)	1.4 ± 1.9 (0–10)
OHIP-G49 sum score	10.5 (4.0/27.5)	17.6 ± 18.5 (0–83)
Visual analogue scale		
Self-perceived esthetic (VASe)	80.5 (61.3/94.4)	76.0 ± 22.5 (20.0–100.0)
Chewing function (VASc)	90.5 (80.0/100.0)	86.3 ± 16.3 (25.0–100.0)
Hygiene ability (VASh)	80.0 (71.3/90.0)	79.8 ± 15.8 (30.0–100.0)

*SD*, standard deviation; *IQR*, interquartile range; *OHIP*, oral health impact profile; *VAS*, visual analogue scale

**Fig. 3:**
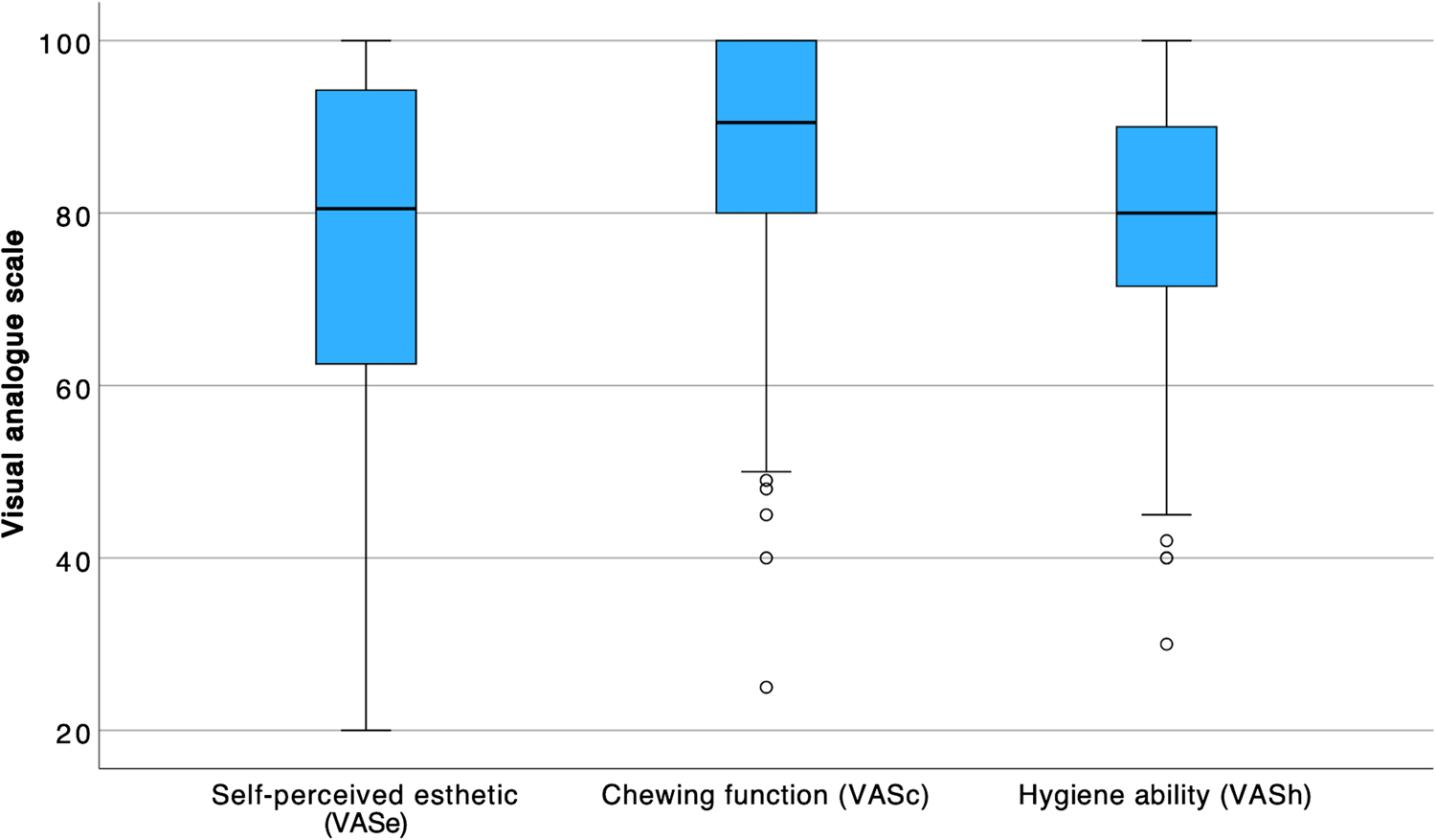
Distribution of VAS ratings (wide black line, median; box, 25–75% range of all values; whiskers, range of all values without outliers; circle, outliers)

On the VAS, in all three categories, the patients indicated values in the upper quarter of the scale (Fig. 3). The mean values were 76.0±22.5 for VASe, 86.3±16.3 for VASc, and 79.8±15.8 for VASh (Table 4).

### Interactions between PROMs/oHRQoL and risk factors

In a linear regression analysis of OHIP-G49 scores and the risk factors previously identified as significant in univariate comparisons, a significantly negative association was shown for the private insurance status (*p*=0.015, *R*^2^=0.204), indicating a higher satisfaction with their oHRQoL (Table 5).

**Table 5:** Linear regression analysis: OHIP-G49 according to different risk factors

Parameter	Regression coefficient	95% CI		SE	*p* value
		Lower limit	Upper limit		
Constant	25.398	12.620	38.177	6.443	< 0.0001
Insurance status (private)	−9.234	−16.609	−1.859	3.718	0.015
Number of SPC	−0.234	−0.711	0.243	0.241	0.333
Compliance with SPC	−5.573	−13.946	2.800	4.222	0.190
Change of therapist (yes)	3.924	−3.440	11.289	3.713	0.293
PCR (T1)	0.085	−0.101	0.271	0.094	0.368

Dependent variable: OHIP-G49 (*n* = 108)

Adjusted *R*^2^ = 0.204

*SPC*, supportive periodontal care; *PCR*, plaque control record; *SE*, standard error; *CI*, confidence interval

Regarding the VAS values, the VASe value showed a significantly positive association with compliant patients (*p*=0.032, *R*^2^=0.204). The VASc value showed both a significantly negative association with active smoking status at T1 (*p*=0.012, *R*^2^=0.084) and a significantly positive association with private insurance (*p*=0.005, *R*^2^=0.084). For the VASh value, a significantly positive association was demonstrated for privately insured patients (*p*=0.012, *R*^2^=0.222), while a significantly negative association was shown for the number of lost molars (*p*=0.008, *R*^2^=0.222) as well as BOP at T1 (*p*=0.029, *R*^2^=0.222) (Table 6).

**Table 6:** Linear regression analysis: VAS according to different risk factors

Parameter	Regression coefficient	95% CI		SE	*p* value
		Lower limit	Upper limit		
(a) VAS self-perceived esthetic					
Constant	72.258	57.003	87.514	7.692	< 0.0001
Insurance status (private)	4.956	−4.540	14.453	4.788	0.303
Number of SPC	−0.103	−0.722	0.516	0.312	0.741
Compliance with SPC	11.928	1.078	22.778	5.471	0.032
Change of therapist (yes)	−4.706	−14.206	4.795	4.790	0.328
(b) VAS chewing function					
Constant	88.315	82.098	94.533	3.135	< 0.0001
Smoking status (T1, active smoker)	−5.260	−9.359	−1.161	2.067	0.012
Insurance status (private)	8.720	2.708	14.732	3.031	0.005
BOP (T1)	−0.353	−0.974	0.268	0.313	0.262
PISA (T1)	0.002	−0.036	0.039	0.019	0.918
(c) VAS hygiene ability					
Constant	70.411	52.787	88.034	8.878	< 0.0001
Age (T1)	0.106	−0.182	0.394	0.145	0.468
Smoking status (T1, active smoker)	−2.847	−6.915	1.222	2.049	0.168
Insurance status (private)	8.294	1.883	14.705	3.230	0.012
Initial diagnosis (severe)	2.624	−1.191	6.440	1.922	0.175
Number of SPC	0.076	−0.356	0.508	0.218	0.727
Compliance with SPC	1.607	−5.765	8.980	3.714	0.666
BOP (T1)	−0.715	−1.354	−0.075	0.322	0.029
Lost molars	−4.189	−7.258	−1.120	1.546	0.008
Antibiotics during APT (yes)	−6.688	−15.299	1.922	4.338	0.126
Surgery during APT (yes)	4.075	−1.753	9.903	2.936	0.168
PISA (T1)	0.032	−0.008	0.072	0.020	0.111

Dependent variable: (a) VAS self-perceived esthetic (*n* = 108), (b) VAS chewing function (*n* = 108), (c) VAS hygiene ability (*n* = 108)

Adjusted *R*^2^ = (a) 0.204, (b) 0.084, (c) 0.222

*VAS*, visual analogue scale; *SPC*, supportive periodontal care; *BOP*, bleeding on probing; *PISA*, periodontal inflamed surface area; *APT*, active periodontal therapy; *SE*, standard error; *CI*, confidence interval

## Discussion

Numerous long-term studies showed that, after completion of APT, patients expressed a high level of satisfaction with their oHRQoL [17–20]. In the present study, patients were asked to evaluate their oHRQoL as well as PROMs 10 years after completion of APT. The mean OHIP sum score after 10 years was 17.6±18.5 (0–83). The worst score (83) was nevertheless 50% lower than the possible maximum score of 196. The highest mean score of 4.4±3.8 was reached in the subscale “functional limitations” and the lowest mean score of 1.2±2.1 in the subscale “social disability.”

Over follow-up periods of 10–20 years after APT, other studies also reported a high degree of satisfaction showing mean OHIP-G49 sum scores between 13.78 and 24.9 in periodontally compromised patients [18–20].

In 63 patients with a previous history of chronic periodontitis [29], a mean OHIP-G49 sum score of 18.89±21.66 was reported after a treatment period of 20 years [18]. Similar to the subscales with respective scores of 4.4±3.8 and 3.9±4.1 indicated in our cohort of patients, the individual subscales showed the highest mean scores of 4.59 for “functional limitations” and of 4.08 for “physical pain.” Other studies showed similar results in their evaluation of the subscales [45–47]. Moreover, privately insured patients (34.9%) showed significantly lower OHIP scores (*p*=0.0021) than patients covered by statutory health insurance, thus indicating a higher satisfaction with their oHRQoL [18]. Using linear regression analysis, the present study also revealed a positive association between the mean OHIP sum score and the private insurance status (*p*=0.015).

In addition, El Sayed et al. (2018) [18] showed that compliance had a positive (*p*=0.04) and active smoking status a negative effect (*p*=0.041) on the mean OHIP score. While 56.4% of our patient cohort complied with the recommended individual SPC interval, this was true for only 15.9% of all patients investigated in the study by El Sayed et al. (2018) [18]. This may explain why in the present study, the lack of compliance was not identified as a risk factor for high OHIP scores, even though the score was significantly higher (*p*<0.0001) in the group of non-compliant patients (25.05±21.58) than in the group of compliant patients (11.9±13.3). The proportion of smokers in our cohort of patients (13.9%) was similar to that reported by El Sayed et al. (2018) [18] (14.3%). However, comparability is limited since the follow-up period in the study by El Sayed et al. (2018) [18] was twice as long as in our patient cohort.

Another study on 71 patients with a history of aggressive periodontitis investigated oHRQoL 5 years after completion of APT and reported a mean OHIP-G49 sum score of 24.9. Also here, it was demonstrated that privately insured patients (14.1%) showed significantly lower OHIP scores than patients covered by statutory health insurance. This confirms the results of the present study (private insurances, 11.37±1.59; statutory health insurance, 22.70±3.01; *p*<0.0001). In a university setting, privately insured patients are often treated by dentists, while patients covered by statutory health insurance tend to be treated in student courses, which, in addition to requiring more time, also implies more frequent changes of therapist. This may explain the discrepancies consistently observed between the group of privately insured patients, on the one hand, and the group of patients covered by statutory health insurance, on the other hand.

In the study of Bäumer et al. (2018) [19], patients adhered to a recommended SPC interval less than half as often as compared to the cohort of patients investigated in the present study (21.2% vs. 56.4%). Patients who regularly attended SPC or were non-smokers showed lower OHIP scores than patients who irregularly attended SPC (*p*=0.0162) or were smokers (*p*=0.0204) [19]. This is in line with the results of our study (compliant patients, 11.9±13.3; non-compliant patients, 25.1±21.5, *p*<0.0001; non-smoker, 16.5±17.3; smoker, 28.4±27.5, *p*=0.051). Further studies confirmed a correlation between regular SPC and oHRQoL [48, 49].

A systematic review as well as other studies reported an association between oHRQoL and the degree of severity of the disease [3, 45, 50]. However, the results of the present study did not confirm this connection probably due to the unequal distribution of stages of disease.

Another study conducted in a practice setting and covering an average follow-up period of 20 years reported a mean OHIP-G49 sum score of 13.78±15.59 (0–70) for 56 periodontally compromised patients [20]. The fact that this score is slightly lower than the score shown in our study may be in part due to less frequent changes of therapist occurring in a practice setting as compared to a university setting.

Patients with untreated periodontitis express a significantly lower degree of satisfaction in comparison to periodontally healthy patients. Durham et al. (2013) [51] reported a mean OHIP sum score of 48.6±32.0 for 89 periodontally compromised patients and a score of 36.8±29.8 for 89 periodontally healthy patients (*p*<0.01) [51]. These data are confirmed by the study of Levin et al. (2018) [52] which also reported significantly worse mean OHIP sum scores (OHIP-14, 10.65±8.47) in a case–control study on 98 patients with untreated chronic periodontitis compared to 48 periodontally healthy controls (OHIP-14, 6.66±5.78) (*p*=0.004) [52].

The study by Junge et al. (2021) [20] indirectly confirmed this finding. In fact, in that study, 51 periodontally healthy/gingivitis patients (OHIP-G49, 12.04±12.18) were followed-up in addition to 56 periodontally compromised but systematically treated patients (OHIP-G49, 13.78±15.59). No significant difference was found between the two groups in terms of mean OHIP sum score (*p*=0.484). According to a systematic review based on ten studies, the probability of a worse OHIP sum score (OHIP-14) increases by a factor of 3.5 in case of untreated severe periodontitis compared to periodontally healthy patients [53].

Surprisingly, in the present study, the mean OHIP sum score of 17.6±18.5 registered in a cohort of periodontally treated patients is only half as high as the score reported in the study by Durham et al. (2013) [51] in periodontally healthy patients (36.84±29.80) [51]. The different origins of the cohorts examined (the UK and Germany) and the respective different health care systems may have an impact on the reported scores. Moreover, the differences in OHIP scores may be attributable to the mean age of the patients (47±9 years) investigated in the study by Durham et al. (2013) [51]. In the present study, the mean age of patients was 65.4±10.7 years at the time of the survey. The higher average age could imply that elderly people tend to adapt themselves to their oral condition. On the other hand, the sense of achievement experienced by patients after a successfully treated chronic disease may also have affected the OHIP sum score, an achievement which is “missing” in periodontally healthy patients. In addition, sociodemographic factors such as the educational status, which was high (66%) in our cohort of patients, may have influenced the oHRQoL [18, 45, 50].

In addition to the OHIP-G49 questionnaire, all patients of our cohort answered three questions by marking a VAS. In all three categories (VASe, 76.0±22.5; VASc, 86.3±16.3; VASh, 79.8±15.8), patients indicated values in the upper quarter of the scale, confirming their high degree of satisfaction revealed in the OHIP questionnaire.

In the study by Junge et al. (2021) [20], the same three VAS questions were asked in addition to the OHIP-G49 questionnaire. In comparison to the present study, Junge et al. (2021) [20] reported an even higher level of satisfaction in periodontally compromised patients (VASe, 81.38±18.57; VASc, 89.91±11.29; VASh, 83.29±15.33) which did not significantly differ from that of the periodontally healthy or gingivitis patients (VASe, 77.88±20.87; VASc, 89.88±12.41; VASh, 82.71±14.81; *p* values, VASe, 0.309; VASc, 0.362; VASh, 0.989) [20]. In particular, it may be hypothesized in the group of periodontally compromised patients that the divergent VAS values are attributable to the three times higher tooth loss rate and an average follow-up period which in the present study is only half as long in the cohort of patients investigated (135 teeth; 0.12 teeth/patient/year) as compared to the patient cohort examined by Junge et al. (2021) [20] (38 teeth; 0.04 teeth/patient/year). Furthermore, in that study, patients were on average about 15 years younger at the time of the survey (49.1±10.9) and had received constant care for many years without frequent changes of therapist, in contrast to the present study which was conducted in a university setting also involving students. The latter circumstance may explain the different tooth loss rates; as in the practice setting, the treatment philosophy based on tooth preservation was more constantly maintained due to less frequent changes of therapist [23, 24].

Linear regression analysis of VAS values for different risk factors showed a significantly positive association between VASe and the patients’ degree of compliance (*p*=0.032). This may be why patients with a strong sense of esthetics are more compliant when it comes to maintaining their sense of well-being.

A significantly negative association was identified between VASc and active smoking status at the start of SPC (*p*=0.012). In fact, smoking is one of the best-established factors for the progression of periodontal disease culminating in tooth loss [54]. In the present study, 15 smokers lost 19 teeth and showed a slightly higher percentage of CAL>5mm (12%) at T2 compared to non-smokers and former smokers (9%). Both tooth loss and increased CAL may affect the assessment of their masticatory function.

Also, there is a significantly positive association between VASc and private insurance status (*p*=0.005). This may have been attributable to prosthetic rehabilitation and the associated costs and their reimbursement. Furthermore, privately insured patients were primarily treated by dentists, while a large proportion of the patients covered by statutory health insurance were treated (subgingival instrumentation and SPC) by students who usually require substantially more time to restore masticatory function.

Regarding VASh, a significantly positive association with private insurance status was detected (*p*=0.012). In fact, after 10 years, privately insured patients had a mean PCR score of 30.53%, while patients covered by statutory health insurance reached a PCR score of 35.65%. However, since VAS values reflect the subjective rather than the objective perception, the mean number of annual SPC appointments (privately insured, 1.8±0.81; patients covered by statutory health insurance, 2.6±0.84) may have influenced this significantly positive association. Moreover, a negative association was identified between VASh and the number of lost molars (*p*=0.008) as well as the BOP value at the start of the SPC (*p*=0.029). An elevated BOP value is an indicator of increased periodontal inflammation which may lead to an increased bleeding tendency during daily oral hygiene and influence the subjective perception [55]. The loss of a molar may result in a prosthesis that, due to its distal position in the oral cavity, is difficult to reach and to clean during oral hygiene.

In the present study, patients with tooth loss tend to show worse OHIP-G49 and VAS scores (OHIP-G49, 18.02; VASe, 73.4; VASc, 84.1; VASh, 77.1) during SPC than those who have not lost any teeth (OHIP-G49, 17.2; VASe, 78.6; VASc, 88.4; VASh, 82.4). A study of a cohort that was also older than 60 years at the time of the follow-up examination was able to demonstrate an average 4.8 point increase in the OHIP-14 value in the case of tooth loss [56]. However, a direct correlation with tooth loss could not be established in the present analysis. Moreover, although the influence of prosthetic restorations on the oHRQoL and patient satisfaction is known, no correlation could be found in the present study [57]. There was a shift from tooth- to implant-supported restorations during SPC, but it did not show any significant influence on the OHIP-G49 and VAS values. Even if the results of this study coincide with those of other studies, it cannot be ruled out that, due to the retrospective study design, basically satisfied patients remained in treatment for more than 10 years after the end of the active therapy phase, whereas dissatisfied patients no longer wanted to be treated in this institution [18–20].

The limitations of the present study can be summarized as follows: (1) OHIP scores and VAS values were collected only at T2 and therefore do not allow any conclusions on the long-term development of oHRQoL. (2) No data are available for three of the OHIP-G49 questions. Answers to those three questions might have led to different results. Nevertheless, the findings of the present study are largely consistent with those described in the already existing literature.

A largely periodontal approach to oHRQoL and PROMs of the patients that does not consider conservative treatments occurred in the meantime, and their effect on oral conditions or possible confounding effects of sociodemographic factors should not lead to an overinterpretation of the data.

## Conclusions

After 10 years of SPC in a university setting, it is possible to obtain satisfactory values for oHRQoL and PROMs in most of the treated patients. The identified factors (private health insurance status, compliance, smoking status, and BOP at the start of SPC as well as the number of lost molars) may help to predict patient satisfaction in the long-term.
